# The IMPACT Survey: the economic impact of osteogenesis imperfecta in adults

**DOI:** 10.1186/s13023-024-03218-6

**Published:** 2024-06-03

**Authors:** Tracy Hart, Ingunn Westerheim, Taco van Welzenis, Oliver Semler, Cathleen Raggio, Frank Rauch, Ruby Dadzie, Samantha Prince, Lena Lande Wekre

**Affiliations:** 1 Osteogenesis Imperfecta Foundation, Gaithersburg, MD USA; 2 Osteogenesis Imperfecta Federation Europe, Heffen, Belgium; 3 Faculty of Medicine and University Hospital Cologne, Department of Paediatrics, University of Cologne, Cologne, Germany; 4 Hospital for Special Surgery, New York, USA; 5 McGill University, Montreal, Canada; 6 Wickenstones Ltd, Abingdon, Oxfordshire UK; 7 TRS National Resource Center for Rare Disorders, Sunnaas Rehabilitation Hospital, Nesodden, Norway

**Keywords:** Osteogenesis imperfecta, Patient reported outcomes, Survey, Burden of disease, Economic burden, Healthcare resource use, Productivity loss, Out-of-pocket spending

## Abstract

**Background:**

The IMPACT survey aimed to elucidate the humanistic, clinical and economic burden of osteogenesis imperfecta (OI) on individuals with OI, their families, caregivers and wider society. Research methodology, demographics and initial insights from the survey have been previously reported. The cost of illness (healthcare resource use, productivity loss, out-of-pocket spending) and drivers of the economic impact of OI are reported here.

**Methods:**

IMPACT was an international mixed-methods online survey in eight languages (fielded July–September 2021) targeting adults (aged ≥ 18 years) or adolescents (aged ≥ 12–17 years) with OI, caregivers with or without OI and other close relatives. Survey domains included demographics, socioeconomic factors, clinical characteristics, treatment patterns, quality of life and health economics. The health economic domain for adults, which included questions on healthcare resource use, productivity loss and out-of-pocket spending, was summarised. Regression and pairwise analyses were conducted to identify independent drivers and associations with respondent characteristics.

**Results:**

Overall, 1,440 adults with OI responded to the survey. Respondents were mostly female (70%) and from Europe (63%) with a median age of 43 years. Within a 12-month period, adults with OI reported visiting a wide range of healthcare professionals. Two-thirds (66%) of adults visited a hospital, and one-third (33%) visited the emergency department. The mean total number of diagnostic tests undergone by adults within these 12 months was 8.0. Adults had undergone a mean total of 11.8 surgeries up to the time point of the survey. The proportions of adults using queried consumables or services over 12 months ranged from 18–82%, depending on the type of consumable or service. Most adults (58%) were in paid employment, of which nearly one-third (29%) reported missing a workday. Of the queried expenses, the mean total out-of-pocket spending in 4 weeks was €191. Respondent characteristics such as female sex, more severe self-reported OI and the experience of fractures were often associated with increased economic burden.

**Conclusion:**

IMPACT provides novel insights into the substantial cost of illness associated with OI on individuals, healthcare systems and society at large. Future analyses will provide insights into country-specific economic impact, humanistic impact and the healthcare journey of individuals with OI.

**Supplementary Information:**

The online version contains supplementary material available at 10.1186/s13023-024-03218-6.

## Introduction

Osteogenesis imperfecta (OI) is a rare, heritable connective tissue disorder with an estimated incidence of 1/15,000–20,000; however, the actual number may be higher [1–4]. It is often a result of mutations in the type 1 collagen genes (*COL1A1* and *COL1A2*), but mutations in other collagen synthesis-related genes are also associated with an OI-like phenotype [2]. OI affects multiple body systems and organs, resulting in an array of secondary features, including skeletal deformities, blue sclerae, hearing loss, dentinogenesis imperfecta, basilar invagination and cardiovascular and pulmonary abnormalities [2]. Currently, no curative therapies for OI exist. Treatments aim to reduce fractures, improve mobility and self-support, and manage other symptoms. These comprehensive care strategies typically require a multidisciplinary team of specialists [5] and include medications such as bisphosphonates [6, 7], human monoclonal antibodies or parathyroid hormone [8, 9], orthopaedic interventions and physical therapy [10]. Due to the complexities of OI and its treatment, the impact on patients and healthcare systems is considerable [11].

To date, reports on the economic implications of OI have been limited, predominantly focusing on children and specific geographical regions [11–22]. These studies have shed light on resource use [12–14, 16–18, 21], direct medical costs, such as treatments and hospitalisations [11–20], and indirect medical costs (out-of-pocket expenses, such as travel expenditures) [12, 20, 22]. However, none explored expenses linked to co-payments (fixed, predetermined costs that individuals pay for specific medical services or prescription drugs as part of their health insurance coverage) or home modifications. The IMPACT Survey was conducted to better understand the economic, as well as the humanistic and clinical, impact of OI on individuals and wider society [23]. Here, we describe the cost of illness associated with OI in adults and identify independent drivers and associations with respondent characteristics.

## Methods

### Development

IMPACT was developed by a steering committee that included academic researchers, representatives of the patient advocacy organisation (PAO) Osteogenesis Imperfecta Foundation (OIF, USA), the umbrella PAO Osteogenesis Imperfecta Federation Europe (OIFE) and representatives of Mereo BioPharma. Evidence gaps in OI literature were identified in a scoping review [24]; topics that were most relevant to the OI and research communities and most suitable to survey-based research were prioritised. The questionnaire was drafted and reviewed in English and professionally translated into German, Italian, Dutch, French, Russian, Spanish (both South American and European) and Portuguese. Translations were localised with the help of PAO members from relevant geographies who advised on regionally relevant answer options and question wording. For more information on the development, design and fielding of IMPACT, please refer to Westerheim et al. [23].

### Survey domains

Survey domains included demographics, socioeconomic factors, clinical characteristics, treatment patterns, quality of life and health economics [23]. For adults, the health economic domain asked about healthcare resource use over the past 12 months and throughout their lifetime. This included the number of visits to various healthcare professionals (HCPs); visits to the hospital, emergency department and rehabilitation centre; the number of diagnostic tests and surgeries; and the use of OI-related consumables (e.g., manual wheelchairs) and services (e.g., dental work). The survey also asked about productivity loss and direct payments individuals made for healthcare costs not covered by their insurance (out-of-pocket spending), such as personal care or support assistance, in the past 4 weeks.

### Data processing

Survey data were imported into Microsoft Excel, translated back into English and compiled into a master database using the pandas Python software package. Excel was used to clean (to exclude any outliers and non-sensical responses), code and validate data, as well as generate descriptive statistics. Potential outliers were identified as any values greater than 2 standard deviations (SD) from the median of continuous variables and validated by co-authors with clinical experience.

### Descriptive analysis

Categorical measures are presented as frequency (number of patients, n) and percentage (%) of total survey respondents. Continuous and count variables are reported as mean and SD.

### Regression and pairwise analyses

Logistic and Poisson regression analyses were conducted to identify independent predictors, henceforth called drivers, of healthcare resource use, productivity loss and out-of-pocket spending (Appendix Tables 1 and 2). Tested drivers included respondent characteristics age, sex and self-reported OI severity, as well as clinical signs, symptoms and events experienced in the past 12 months. The variable for the clinical symptom “gynaecological problems” was omitted from the regression models due to collinearity with the variable for sex. Outcomes of the regression analyses are reported as either the incidence rate ratio (IRR) or odds ratio (OR).

To supplement the analysis of drivers, a pairwise analysis was performed to identify associations between healthcare resource use, productivity loss, out-of-pocket spending and respondent characteristics (Appendix Table 3). To test for differences in sample proportions within categorical and continuous variables, chi-squared test and Student’s t-test (equal and unequal variance) were performed as appropriate.

Regression and pairwise analyses were performed using Stata, Version 15.1. *P* values ≤ 0.05 were considered statistically significant.

## Results

### Demographics

Overall, 1,440 adults with OI responded to the survey. Respondents were mostly female (70%) and from Europe (63%) with a median age of 43 years (range 18–85). As previously reported [23], most adults rated their OI as moderate (47%), while the smallest proportion rated their OI as severe (14%). Similarly, the majority of adults reported clinical OI type 1 (38%), 3 (16%) and 4 (11%) (Table 1; [23]). Examining the relationship between clinical OI type and self-reported OI severity revealed a broad alignment (Appendix Tables 4 and 5; [23]). Further details, including a breakdown by geographic region and employment status, are reported in Appendix Tables 4 and 5.

**Table 1:** Demographics

	**Adults with OI (*n* = 1,440)**	**Male participants**^**a,b**^** (*n* = 423)**	**Female participants**^**a,b**^** (*n* = 1,008)**
**Age, mean (range)**^**a,c**^	43.3 (18–85)	43.0 (18–83)	43.6 (18–85)
**Geography, N (%)**^**a,d**^			
Europe	911 (63.3)	279 (66.0)	625 (62.0)
North America	347 (24.1)	87 (20.6)	259 (25.7)
South America	61 (4.2)	19 (4.5)	42 (4.2)
Asia	78 (5.4)	31 (7.3)	46 (4.6)
Africa	7 (0.5)	3 (0.7)	4 (0.4)
Australia/Oceania	36 (2.5)	4 (1.0)	32 (3.2)
**OI severity, N (%)**^**a,e**^			
Mild	507 (35.2)	137 (32.4)	366 (36.3)
Moderate	671 (46.6)	205 (48.5)	461 (45.7)
Severe	205 (14.2)	65 (15.4)	140 (13.9)
I don’t know	53 (3.7)	15 (3.6)	38 (3.8)
Prefer not to say	4 (0.3)	1 (0.2)	3 (0.3)
**OI type, N (%)**^**a,f**^			
Undefined type	127 (8.8)	33 (7.8)	92 (9.1)
Type 1 (I)	543 (37.7)	142 (33.6)	399 (39.6)
Type 2 (II)	23 (1.6)	9 (2.1)	14 (1.4)
Type 3 (III)	225 (15.6)	76 (18)	146 (14.5)
Type 4 (IV)	158 (11.0)	43 (10.2)	114 (11.3)
Type 5 (V)	26 (1.8)	6 (1.4)	20 (2.0)
Type 6 (VI)	4 (0.3)	2 (0.5)	2 (0.2)
Type 7 (VII)	2 (0.1)	1 (0.2)	1 (0.1)
Type 8 (VIII)	1 (0.1)	1 (0.2)	0 (0.0)
Type 9 (IX)	1 (0.1)	1 (0.2)	0 (0.0)
Type 10 (X)	0 (0.0)	0 (0.0)	0 (0.0)
Type 11 (XI)	2 (0.1)	0 (0.0)	2 (0.2)
Type 12 (XII)	0 (0.0)	0 (0.0)	0 (0.0)
Type 13 (XIII)	0 (0.0)	0 (0.0)	0 (0.0)
Type 14 (XIV)	0 (0.0)	0 (0.0)	0 (0.0)
Type 15 (XV)	1 (0.1)	0 (0.0)	1 (0.1)
Type 16 (XVI)	0 (0.0)	0 (0.0)	0 (0.0)
Type 17 (XVII)	0 (0.0)	0 (0.0)	0 (0.0)
Type 18 (XVIII)	0 (0.0)	0 (0.0)	0 (0.0)
Other	39 (2.7)	12 (2.8)	27 (2.7)
I don’t know	286 (19.9)	96 (22.7)	189 (18.8)
Prefer not to say	2 (0.1)	1 (0.2)	1 (0.1)
**Employment status, N (%)**^**g**^			
Not in paid employment	603 (41.9)	156 (36.9)	440 (43.7)
Employed full-time	491 (34.1)	177 (41.8)	313 (31.1)
Employed part-time	230 (16.0)	48 (11.4)	182 (18.1)
Self-employed	104 (7.2)	41 (9.7)	62 (6.2)
Other^h^	7 (0.5)	0 (0.0)	7 (0.7)
Prefer not to say	5 (0.4)	1 (0.2)	4 (0.4)

*Abbreviation*: *OI* Osteogenesis imperfecta

^a^Also reported in Westerheim et al. [23]

^b^Question 8 “What is your sex?”

^c^Question 1 “What is your age?”

^d^Question 7 “What is your country of residence?”

^e^Question 18 “How would you describe the severity of your OI?”

^f^Question 17 “If you have received an OI type as part of your OI diagnosis or treatment, please indicate your type using the dropdown below”

^g^Question 9 and 10 “Please indicate which of the following best describe you/What is your current paid employment status?”

^h^‘Other’ includes respondents who were in paid full-time internships or paid jobs but were not working at the time due to a leave of absence

### Healthcare resource use

**Fig. 1:**
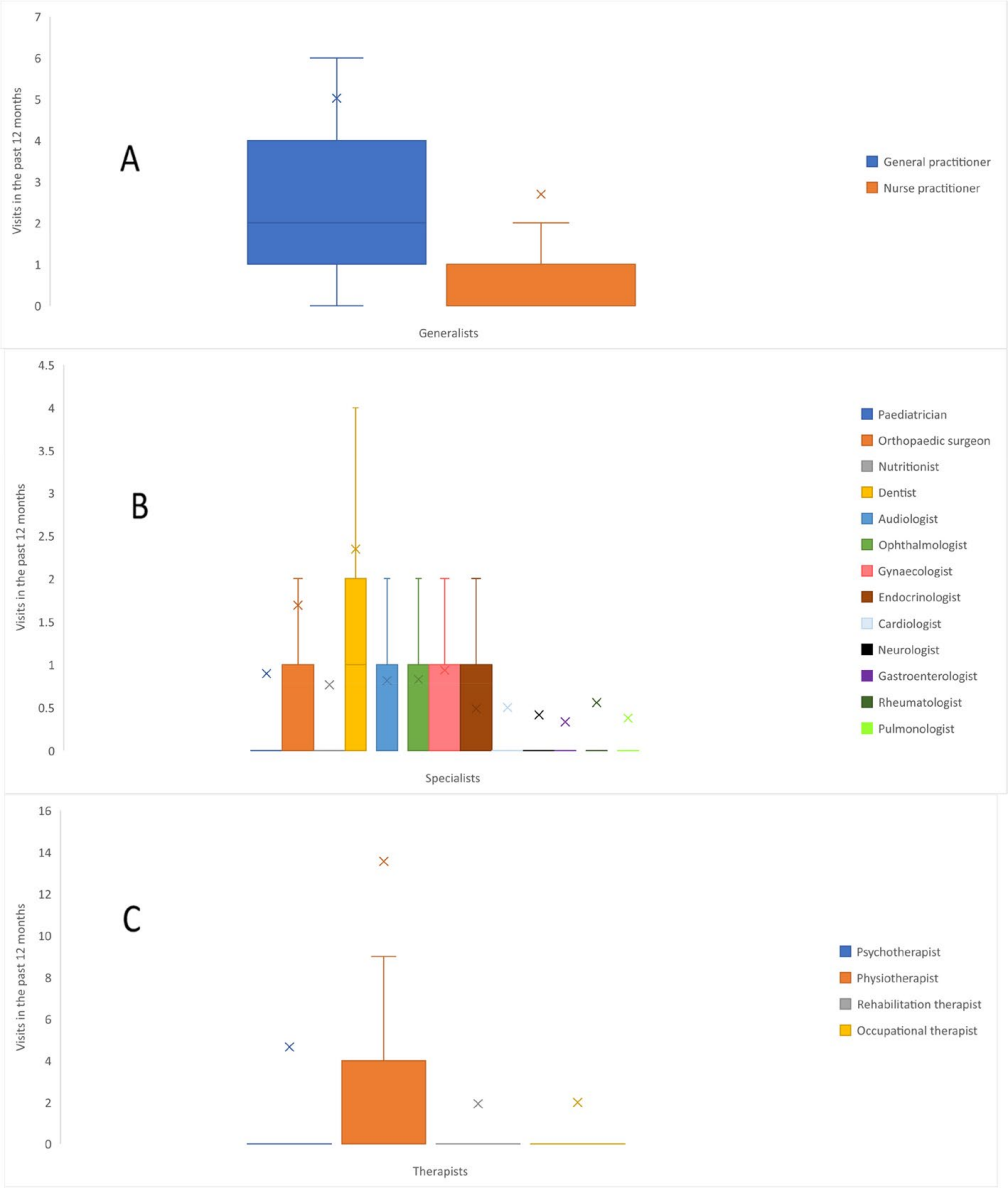
Visits to **A** generalist, **B** specialist and **C** therapist HCPs in the past 12 months ^a^. Abbreviations: HCP, healthcare professional. Footnotes: ^a^ Box plot elements: Minimum: The lower end of the whisker represents the minimum value in the dataset, excluding outliers; First Quartile (Q1): The bottom edge of the box represents the first quartile, which is the value below which 25% of the data falls; Median (Q2): The horizontal line within the box represents the median, which is the middle value in the dataset when sorted in ascending order. It divides the data into two equal halves; Third Quartile (Q3): The top edge of the box represents the third quartile, which is the value below which 75% of the data falls; Maximum: The upper end of the whisker represents the maximum value in the dataset, excluding outliers; Interquartile Range (IQR): The length of the box, defined by the distance between the first quartile (Q1) and the third quartile (Q3), represents the interquartile range. It measures the spread of the central 50% of the data; Whiskers: The vertical lines extending from the box represent the range of values that fall within a certain distance from the quartiles. The specific range is often defined as 1.5 times the IQR. Data points beyond the whiskers are considered outliers; Mean: The ‘x’ represents the mean, which is the average value of the dataset and is a measure of the central tendency

Within a 12-month period, adults with OI reported visiting a wide range of HCPs (a mean total of 40.5 visits). These included visits to generalists, such as family doctors and nurse practitioners (mean total 7.7 visits); specialists, such as rheumatologists and neurologists (mean total 10.7 visits); and therapists, such as occupational and rehabilitation therapists (mean total 22.2 visits). Among these, the most frequently visited generalists were general practitioners/family doctors (mean 5.0 visits); the most frequently visited specialists were dentists/orthodontists (mean 2.3 visits); and the most frequently visited therapists were physiotherapists (mean 13.6 visits; Table 2 and Fig. 1A–C).

**Table 2:** Healthcare resource use, productivity loss and out-of-pocket spending related to OI during a given timeframe

**Healthcare professionals, mean visits in the past 12 months (SD)**^**a**^	
Any healthcare professional (including generalists, therapists and specialists)	40.5 (78.1)
Any healthcare professional (excluding therapists)	18.4 (47.2)
**Generalists, mean visits in the past 12 months (SD)**^**a**^	
Any generalist	7.7 (18.1)
General practitioner/family doctor	5.0 (11.3)
Nurse practitioner/care coordinator	2.7 (12.1)
**Specialists, mean visits in the past 12 months (SD)**^**a**^	
Any specialist	10.7 (41.6)
Dentist/orthodontist	2.3 (7.3)
Orthopaedic surgeon/orthopaedist	1.7 (5.9)
Paediatrician	0.9 (8.4)
Nutritionist	0.8 (6.3)
Audiologist	0.8 (5.3)
Ophthalmologist	0.8 (4.7)
Gynaecologist/obstetrician	0.9 (2.3)
Rheumatologist	0.6 (5.2)
Endocrinologist	0.5 (1.1)
Cardiologist	0.5 (5.0)
Neurologist	0.4 (5.9)
Pulmonologist	0.4 (4.2)
Gastroenterologist	0.3 (4.2)
**Therapists, mean visits in the past 12 months (SD)**^**a**^	
Any therapist	22.2 (45.3)
Physiotherapist	13.6 (30.8)
Psychotherapist/counsellor	4.7 (14.8)
Occupational therapist (helps to recover, improve and maintain skills needed for daily living and working)	2.0 (12.5)
Rehabilitation therapist/doctor	1.9 (12.9)
**Hospital and in-patient care, mean visits in the past 12 months (SD)**	
Hospital visits^b^	3.7 (7.4)
Emergency visits^c^	0.8 (3.1)
Nights in hospital^d^	1.2 (6.1)
Rehab nights^e^	1.2 (7.6)
**Diagnostic tests, mean tests in the past 12 months (SD)**^**f**^	
Any diagnostic test	8.0 (10.0)
Blood test	2.5 (4.0)
X-ray	1.9 (3.2)
Urine test	1.1 (1.9)
Ultrasound scan	0.7 (1.8)
Computerised tomography scan	0.4 (1.1)
Magnetic resonance imaging scan	0.4 (0.8)
Audiology test	0.4 (0.8)
Bone density scan	0.3 (0.6)
Echocardiogram	0.3 (0.9)
**Surgeries, mean surgeries in an individual’s lifetime (SD)**^**g**^	
Any surgery	11.8 (15.1)
Fracture repairs	5.6 (9.5)
Rodding	3.2 (6.2)
Dental	1.7 (4.0)
Soft tissue	0.6 (2.4)
Spine	0.3 (1.0)
Hearing	0.3 (1.1)
Basilar invagination	0.1 (0.5)
Heart	0.0 (0.3)
**OI consumable and service use in the past 12 months, n (%)**^**h**^	
Dental work	1177 (81.7)
Manual wheelchair	653 (45.4)
Walking aids	649 (45.1)
Home modifications	646 (44.9)
Vehicle modifications	558 (38.8)
Personal care/support assistance	515 (35.8)
Hearing aids	474 (32.9)
Work modifications	384 (26.7)
Powered wheelchair	370 (25.7)
Breathing aid	263 (18.3)
**Productivity loss, mean workdays missed in the past 4 weeks (SD)**^**i,j**^	
Missed workdays	1.7 (5.3)
**Out-of-pocket spending, mean spend in the past 4 weeks (SD) in Euros**^**k,l,m**^	
**Global (*n* = 1,426)**	
Total spend of queried categories	191.0 (1392.1)
Medicine	40.5 (147.5)
Physiotherapy	29.5 (85.1)
Psychotherapy	12.2 (62.5)
Travel to medical appointments	25.0 (90.5)
Personal care/support assistance	83.8 (1346.1)
**Europe (*n* = 911)**	
Total spend of queried categories	136.5 (336.1)
Medicine	30.3 (68.6)
Physiotherapy	33.0 (84.7)
Psychotherapy	12.8 (69.0)
Travel to medical appointments	25.8 (75.9)
Personal care/support assistance	34.6 (230.0)
**North America (*n* = 347)**	
Total spend of queried categories	285.8 (2300.7)
Medicine	59.4 (158.5)
Physiotherapy	29.2 (101.2)
Psychotherapy	13.7 (57.0)
Travel to medical appointments	29.2 (134.6)
Personal care/support assistance	154.1 (2253.0)
**South America (*n* = 47)**	
Total spend of queried categories	57.3 (71.8)
Medicine	21.2 (22.9)
Physiotherapy	12.8 (29.5)
Psychotherapy	7.5 (21.8)
Travel to medical appointments	9.2 (17.1)
Personal care/support assistance	6.6 (21.8)
**Asia (*n* = 78)**	
Total spend of queried categories	548.2 (3242.5)
Medicine	89.8 (476.2)
Physiotherapy	3.4 (14.1)
Psychotherapy	5.6 (27.7)
Travel to medical appointments	14.5 (32.1)
Personal care/support assistance	434.8 (3148.5)
**Australia/Oceania (*n* = 36)**	
Total spend of queried categories	86.7 (102.2)
Medicine	38.8 (53.7)
Physiotherapy	26.2 (55.1)
Psychotherapy	3.5 (21.0)
Travel to medical appointments	8.7 (20.4)
Personal care/support assistance	9.5 (28.2)

*Abbreviations*: *OI* Osteogenesis imperfecta, *SD* Standard deviation

^a^Question 149 “Please indicate how often you have visited the following healthcare professionals in the past 12 months”

^b^Question 144 “In the past 12 months, how many times have you visited the hospital?”

^c^Question 145 “Of these times (in the past 12 months), how many times have you visited the emergency department?”

^d^Question 146 “In the past 12 months, how many nights did you spend in hospital overall (for both planned and emergency visits)?”

^e^Question 147 “In the past 12 months, how many nights did you spend in a rehabilitation facility?”

^f^Question 148 “In the past 12 months, how many times have you received the following diagnostic tests?”

^g^Question 157 “In your life, how many surgeries have you had for the following things?”

^h^Questions 169 and 178 “If you use any of the following things, who covers the cost?/Do you require any of the following?”

^i^Questions 162 and 173 “In the past 4 weeks, how many days of work have you missed because of your/your child’s or children’s OI?”

^j^The mean number of workdays missed was calculated from respondents in paid employment for whom data were available (*n* = 808). Missed workdays of ‘less than 1’ was estimated as 0.5 days

^k^Questions 170 and 179 “In the past 4 weeks, how much have you spent out of pocket (using your own money) on the following things for yourself and your child/children (with OI)?”

^l^Out-of-pocket costs were converted into Euros (€) using the conversion rate in effect on July 1, 2021

^m^Respondents who indicated their use of Chilean peso (CLF) were excluded from the analysis due to complexities arising from the unusual currency conversion rate

**Fig. 2:**
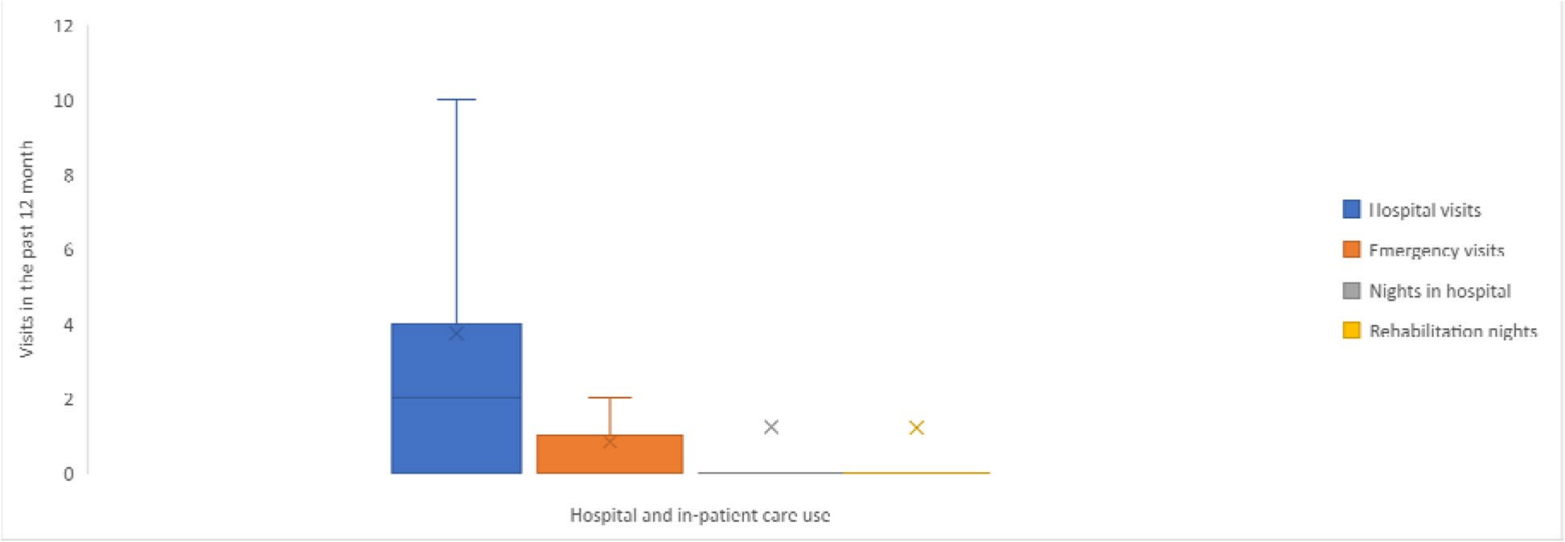
Frequency of hospital and in-patient care use in the past 12 months ^a,b,c,d,e^. Footnotes: ^a^ Box plot elements: Minimum: The lower end of the whisker represents the minimum value in the dataset, excluding outliers; First Quartile (Q1): The bottom edge of the box represents the first quartile, which is the value below which 25% of the data falls; Median (Q2): The horizontal line within the box represents the median, which is the middle value in the dataset when sorted in ascending order. It divides the data into two equal halves; Third Quartile (Q3): The top edge of the box represents the third quartile, which is the value below which 75% of the data falls; Maximum: The upper end of the whisker represents the maximum value in the dataset, excluding outliers; Interquartile Range (IQR): The length of the box, defined by the distance between the first quartile (Q1) and the third quartile (Q3), represents the interquartile range. It measures the spread of the central 50% of the data; Whiskers: The vertical lines extending from the box represent the range of values that fall within a certain distance from the quartiles. The specific range is often defined as 1.5 times the IQR. Data points beyond the whiskers are considered outliers; Mean: The ‘x’ represents the mean, which is the average value of the dataset and is a measure of the central tendency; ^b^ All respondents were asked about their hospital visits in the past 12 months; ^c^ Emergency department visits were only asked to respondents who reported visits to the hospital in the past 12 months; ^d^ The number of nights spent in hospital was only asked of respondents who reported visiting the emergency department in the past 12 months; ^e^ All respondents were asked about how many nights they spent in hospital in the past 12 months

Within a 12-month period, two-thirds of adults (66%) visited a hospital (mean 3.7 visits), and one-third (33%) visited the emergency department (mean 0.8 visits). A considerable proportion reported spending at least one night in hospital (14%) or rehabilitation (17%; mean 1.2 visits for both; Table 2 and Fig. 2).

**Fig. 3:**
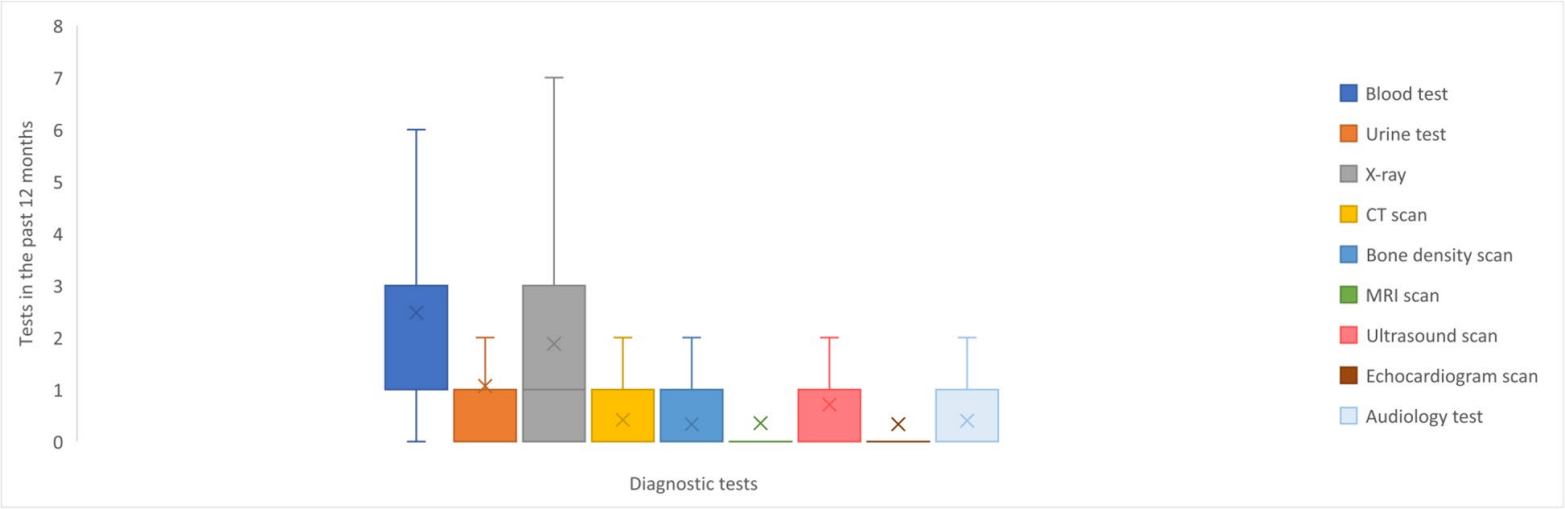
Frequency of diagnostic tests in the past 12 months ^a^. Abbreviations: CT, computerised tomography scan; MRI, magnetic resonance imaging. Footnotes: ^a^ Box plot elements: Minimum: The lower end of the whisker represents the minimum value in the dataset, excluding outliers; First Quartile (Q1): The bottom edge of the box represents the first quartile, which is the value below which 25% of the data falls; Median (Q2): The horizontal line within the box represents the median, which is the middle value in the dataset when sorted in ascending order. It divides the data into two equal halves; Third Quartile (Q3): The top edge of the box represents the third quartile, which is the value below which 75% of the data falls; Maximum: The upper end of the whisker represents the maximum value in the dataset, excluding outliers; Interquartile Range (IQR): The length of the box, defined by the distance between the first quartile (Q1) and the third quartile (Q3), represents the interquartile range. It measures the spread of the central 50% of the data; Whiskers: The vertical lines extending from the box represent the range of values that fall within a certain distance from the quartiles. The specific range is often defined as 1.5 times the IQR. Data points beyond the whiskers are considered outliers; Mean: The ‘x’ represents the mean, which is the average value of the dataset and is a measure of the central tendency

Within a 12-month period, adults underwent a mean total of 8.0 diagnostic tests, with blood tests (mean 2.6 tests), X-rays (mean 1.9 tests), and urine tests (mean 1.1 tests) being the most frequent (Table 2 and Fig. 3).

**Fig. 4:**
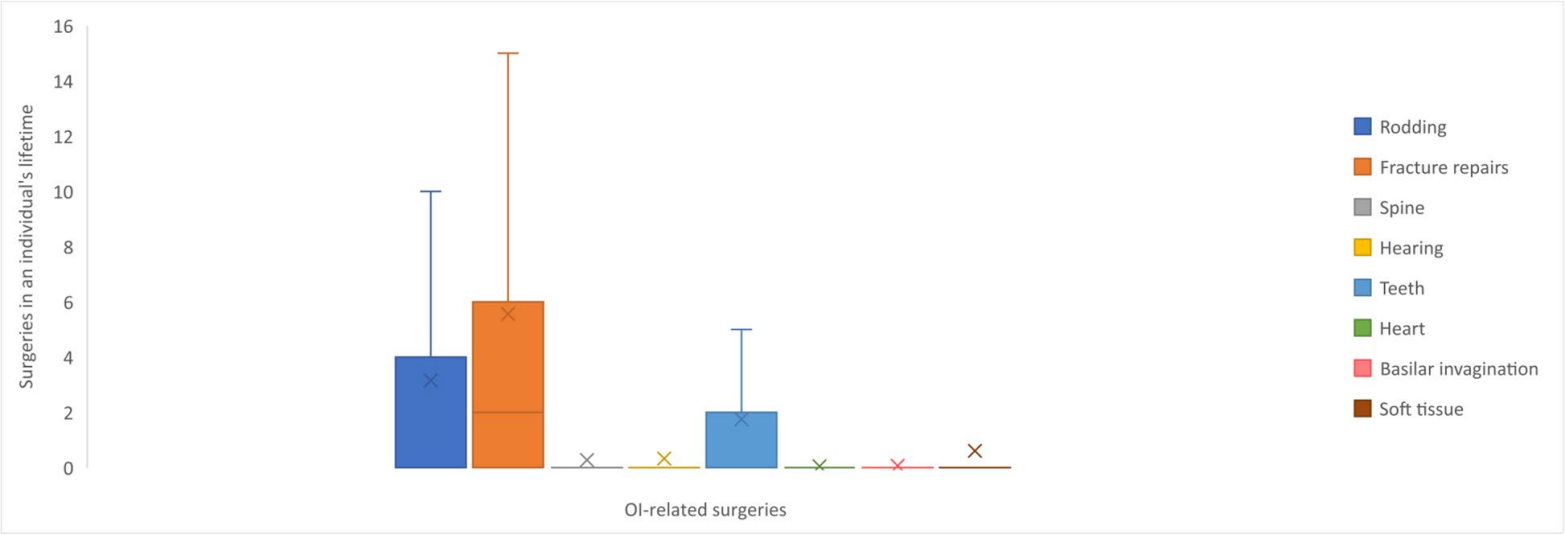
Frequency of surgeries in an individual’s lifetime ^a^. Footnotes: ^a^ Box plot elements: Minimum: The lower end of the whisker represents the minimum value in the dataset, excluding outliers; First Quartile (Q1): The bottom edge of the box represents the first quartile, which is the value below which 25% of the data falls; Median (Q2): The horizontal line within the box represents the median, which is the middle value in the dataset when sorted in ascending order. It divides the data into two equal halves; Third Quartile (Q3): The top edge of the box represents the third quartile, which is the value below which 75% of the data falls; Maximum: The upper end of the whisker represents the maximum value in the dataset, excluding outliers; Interquartile Range (IQR): The length of the box, defined by the distance between the first quartile (Q1) and the third quartile (Q3), represents the interquartile range. It measures the spread of the central 50% of the data; Whiskers: The vertical lines extending from the box represent the range of values that fall within a certain distance from the quartiles. The specific range is often defined as 1.5 times the IQR. Data points beyond the whiskers are considered outliers; Mean: The ‘x’ represents the mean, which is the average value of the dataset and is a measure of the central tendency

Up until the time of the survey, adults with OI had undergone a mean total of 11.8 surgeries. The most common were fracture repairs (mean 5.6 surgeries) and rodding surgeries (mean 3.2 surgeries) while surgeries related to basilar invagination (mean 0.1 surgeries) and the heart (mean 0 surgeries) were the least common (Table 2 and Fig. 4).

**Fig. 5:**
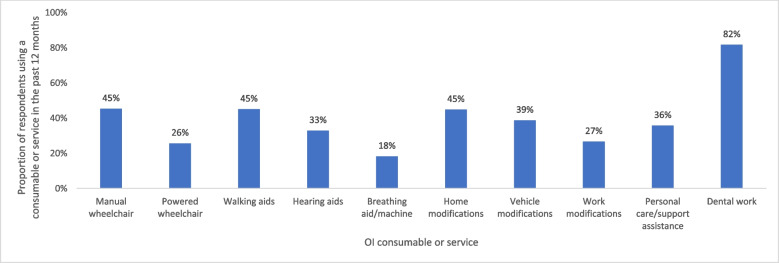
Proportion of respondents reporting consumables or services use in the past 12 months

Within a 12-month period, the proportion of adults using queried consumables or services ranged from 18–82%, depending on the type of consumable or service. Dental work was the service used by the highest proportion of adults (82%); manual wheelchairs, walking aids and home modifications were used by equal proportions of individuals (45% for each; Table 2 and Fig. 5).

### Drivers of healthcare resource use

Adults with self-reported moderate or severe OI reported higher resource use when compared with adults with mild OI. For instance, adults with moderate (IRR 1.7, *P* < 0.01) and severe (IRR 2.8, *P* < 0.01) OI were more likely to visit a physiotherapist within a 12-month period than those with mild OI. Exceptions were observed in visits to orthopaedic surgeons, neurologists, hospitals and ERs, where individuals with mild OI reported higher resource use (Appendix Tables 6 and 7, Appendix Figure 1A–E).

Various clinical signs, symptoms and events were associated with higher resource use. For example, individuals who experienced pain (IRR 2.6, *P* < 0.01) or leg fractures (IRR 4.7, *P* < 0.01) were more likely to spend a night in the hospital within a 12-month period compared with those without (Appendix Tables 6 and 7, Appendix Figure 2A–G).

Female respondents more frequently reported higher resource use when compared with male respondents. For example, within a 12-month period, female respondents were 2.0 (IRR, *P* < 0.01) times more likely to visit a neurologist (Appendix Tables 6 and 7, Appendix Figure 3A–E).

No consistent relationships in resource use were noted across age groups. For instance, while respondents aged 41–50 years were 6.0 (IRR, *P* < 0.01) times more likely to visit a nutritionist within a 12-month period when compared with 18- to 30-year-olds, they were 0.4 (IRR, *P* < 0.01) times as likely to visit a dentist (Appendix Tables 6 and 7, Appendix Figure 4A–E).

### Productivity loss

Most adults with OI were in paid employment (58%; 34% employed full-time, 16% part-time, 7% self-employed and 1% in paid full-time internships or on sick leave from their paid positions). A substantial proportion (15%) were in early retirement due to their disability, and some (2%) faced challenges securing employment.

Within a 4-week period, nearly one-third (29%) of adults in paid employment reported missing workdays (mean 1.7 days missed; Table 2). Notably, one-third (33%) of adults expressed concerns about potential job loss.

### Drivers of productivity loss

Respondents with self-reported moderate (IRR 2.3, *P* < 0.01) and severe (IRR 1.8, *P* < 0.01) OI were more likely to miss a day of work than individuals with mild OI (Appendix Tables 6 and 7, Appendix Figure 1F).

Various clinical signs, symptoms and events, such as fractures (excluding vertebral fractures), were associated with increased productivity loss. For instance, respondents who had experienced at least one arm fracture were 1.9 (IRR, *P* < 0.01) times more likely to miss a workday compared with those who had not (Appendix Tables 6 and 7, Appendix Figure 2H).

Female participants were 1.4 (IRR, *P* < 0.01) times more likely to miss a workday compared with male participants (Appendix Tables 6 and 7, Appendix Figure 3F).

Individuals aged 18–30 years missed fewer workdays than other age groups. For instance, adults aged 51–60 years were 1.7 (IRR, *P* < 0.01) times more likely to miss a workday compared with those aged 18–30 years (Appendix Tables 6 and 7, Appendix Figure 4F).

### Out-of-pocket spending

**Fig. 6:**
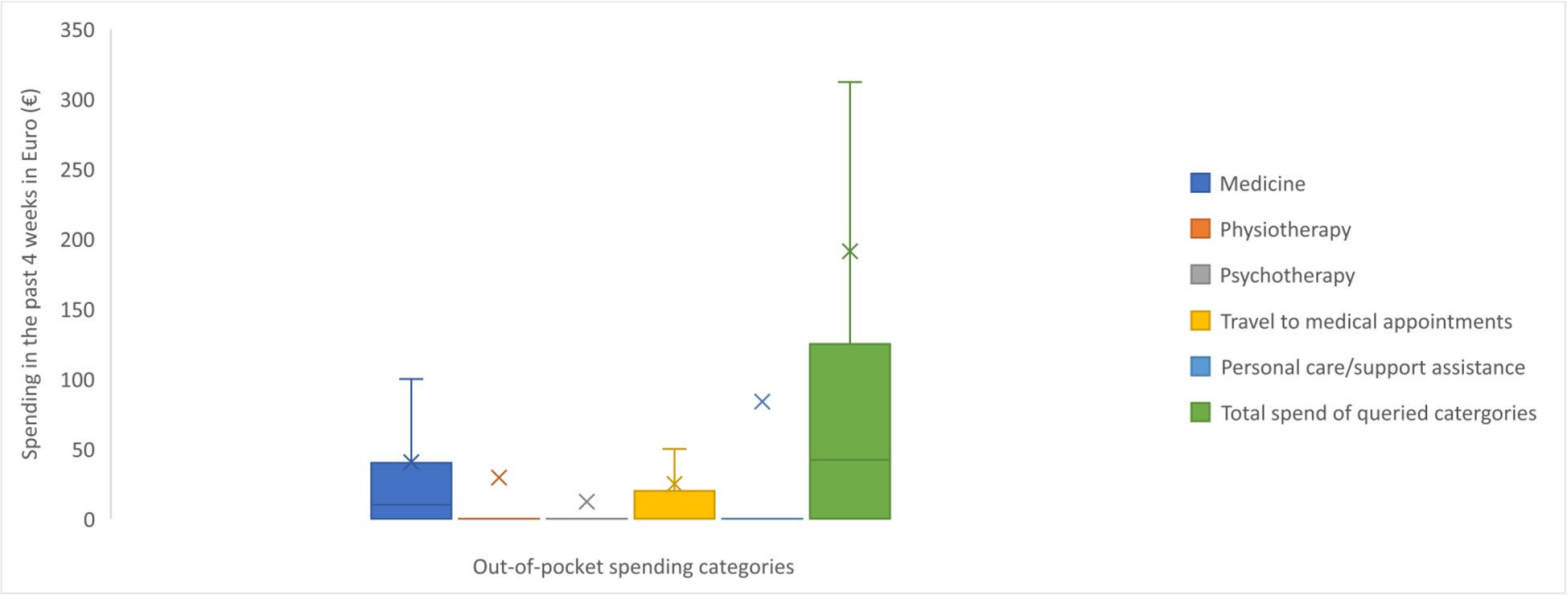
Out-of-pocket spending in the past 4 weeks ^a,b,c^. Footnotes: ^a^ Out-of-pocket costs were converted into Euros (€) using the conversion rate in effect on July 1, 2021; ^b^ Respondents who indicated their use of Chilean peso (CLF) were excluded from the analysis due to complexities arising from the unusual currency conversion rate; ^c^ Box plot elements: Minimum: The lower end of the whisker represents the minimum value in the dataset, excluding outliers; First Quartile (Q1): The bottom edge of the box represents the first quartile, which is the value below which 25% of the data falls; Median (Q2): The horizontal line within the box represents the median, which is the middle value in the dataset when sorted in ascending order. It divides the data into two equal halves; Third Quartile (Q3): The top edge of the box represents the third quartile, which is the value below which 75% of the data falls; Maximum: The upper end of the whisker represents the maximum value in the dataset, excluding outliers; Interquartile Range (IQR): The length of the box, defined by the distance between the first quartile (Q1) and the third quartile (Q3), represents the interquartile range. It measures the spread of the central 50% of the data; Whiskers: The vertical lines extending from the box represent the range of values that fall within a certain distance from the quartiles. The specific range is often defined as 1.5 times the IQR. Data points beyond the whiskers are considered outliers; Mean: The ‘x’ represents the mean, which is the average value of the dataset and is a measure of the central tendency

Of the queried expenses, adults with OI spent a mean total of €191 (range €0–€42,292) out-of-pocket over 4 weeks (Table 2 and Fig. 6). Personal care or support assistance emerged as the category on which respondents spent the most.

Notably, almost two-thirds (64%) of adults with OI expressed concerns about their future financial circumstances.

### Drivers of out-of-pocket spending

Adults with self-reported moderate or severe OI consistently spent more out-of-pocket when compared with adults with mild OI. For instance, individuals with self-reported moderate (IRR 27.9, *P* < 0.01) and severe (IRR 7.5, *P* < 0.01) OI were notably more likely to incur personal care or support assistance expenses compared with those with mild OI (Appendix Tables 6 and 7, Appendix Figure 1G).

Various clinical signs, symptoms and events were associated with higher out-of-pocket expenses. Remarkably, experiencing a fracture was the factor most strongly associated with a higher likelihood of spending on personal care or support assistance. Individuals who had fractured were 8.0 (IRR, *P* < 0.01) times more likely to incur these costs compared with those who had not (Appendix Tables 6 and 7, Appendix Figure 2I).

Female participants consistently reported higher out-of-pocket expenses than male participants. For example, female participants were 4.3 (IRR, *P* < 0.01) times more likely to incur personal care or support assistance expenses (Appendix Tables 6 and 7, Appendix Figure 3F).

No consistent relationships in out-of-pocket spending were noted across age groups. For instance, while individuals aged 51–60 years were 41.1 (IRR, *P* < 0.01) times more likely to incur personal care expenses compared with 18- to 30-year-olds, they were 0.8 (IRR, *P* < 0.01) times as likely to spend money on travel to medical appointments (Appendix Tables 6 and 7, Appendix Figure 4G).

### Pairwise analyses supplementing analysis of drivers

The pairwise analyses revealed multiple factors associated with resource use (Appendix Tables 8–14), productivity loss (Appendix Table 15) and out-of-pocket spending (Appendix Table 16), most of which were consistent with the results of regression analysis. Inconsistencies are highlighted in Appendix Figures 1–4.

### Impact of coronavirus disease (COVID-19)

The COVID-19 pandemic had a significant impact on the healthcare-seeking behaviour of adults with OI and may have resulted in a notable decrease in healthcare resource utilisation reported in the IMPACT survey. In the 12 months prior to survey fielding (1 July–30 September 2021), a substantial proportion of adults with OI reported not only visiting fewer healthcare providers (59%) but also receiving fewer medical diagnostic tests (54%) compared with their usual patterns. Moreover, half of the respondents (50%) reported a shift to predominantly online appointments, and a notable proportion (41%) reported actively avoiding seeking medical care during this period.

## Discussion

With data compiled from 1,440 adults across 66 countries, the IMPACT survey is the most extensive patient-reported dataset on the experience of individuals with OI to date [23]. This survey provides novel insights into the substantial cost of illness associated with OI at the individual level. Most adults experience both direct and indirect costs, which may be exacerbated by demographic and clinical characteristics such as sex, OI severity and the experience of fractures.

Over a 12-month period, adults with OI reported the use of a wide range of healthcare resources, irrespective of their reported characteristics. Our study sheds light on the difference in healthcare resource use between individuals with OI and the general population. For instance, in France in 2021, the average number of doctors’ visits (including generalists and specialists) was 5.5 [25]. In contrast, IMPACT adults with OI had well over three times this number (mean 18.4 visits for the overall adult population and 15.6 for the French population). In another example from France, the average number of annual dental visits (in 2021) was 1.6 [26] compared with the 2.3 in the survey’s overall population and 2.0 in the survey’s French population of adults with OI. This increased healthcare resource use is further evident regarding the frequency of diagnostic tests taken by an individual. For example, in 2021, the mean number of magnetic resonance imaging (MRI) scans among individuals in France, Italy and the United States was 0.1 in each of these countries [27]. Conversely, adults with OI surpassed this number fourfold (mean 0.4 scans), emphasising the increased healthcare resource use associated with OI. While substantial in individuals with OI compared with the general population, healthcare resource use varied within the OI population. For example, adults with self-reported moderate OI had more frequent MRI scans (mean 0.4 scans) than those with mild and severe OI (mean 0.3 scans for both). Our study highlights that at any severity, the healthcare requirements of individuals with OI appear to be higher compared with the general population.

IMPACT serves as a testament to the considerable challenges faced by individuals with OI when seeking employment. In 2021, a significant disparity in unemployment rates was observed when comparing the general population in Germany (3.6%), the United Kingdom (UK; 4.5%) and Spain (14.8%) [28] with individuals with OI in our study (41.9%). Furthermore, our study sheds light on a crucial aspect of this employment disparity: the number of workdays missed. Data derived from the 2021 UK census revealed that, on average, an employee missed 4.6 workdays due to sickness or injury per year [29]. In contrast, our study, covering a 4-week period, revealed that individuals with OI missed an average of 20.4 workdays annually: nearly five times higher than the national average in the UK. While the mean productivity loss in the OI community is substantial, some individuals miss even more workdays. For instance, our study revealed that individuals who had experienced at least one arm fracture missed 5.3 workdays in a month, exceeding the UK national average for a year. This substantial difference highlights the unique challenges faced by those with OI in maintaining consistent work attendance. However, it is important to acknowledge that factors such as workplace support structures, culture and inclusivity, which were not directly explored in our survey, could have influenced this disparity in missed workdays. External factors like the impact of COVID-19 may have also played a role. The increased productivity loss among individuals with OI may be attributed to various factors associated with this chronic condition: the effects of pain, fractures, limited mobility, regular medical appointments and unexpected health complications may affect individuals’ ability to attend work consistently. As well as highlighting the increased productivity loss associated with OI, our study stresses the need for targeted strategies to enhance employment opportunities and provide workplace support for individuals with OI. These measures are essential for fostering a more inclusive and accommodating work environment that caters to the distinctive needs of individuals with OI.

The financial strain experienced by the OI community due to various living expenses is a matter of concern for community members. Our survey revealed that adults with OI may face substantial out-of-pocket expenses, irrespective of their sex, age, OI severity or clinical signs, symptoms and events experienced. While we have highlighted this burden, we acknowledge that our survey’s categorisation of expenses may not have captured the full spectrum of their financial challenges. Other studies have highlighted that financial strain arises from a spectrum of expenditures including home adaptations, treatment costs, lost income and unforeseen hospitalisations [22, 30, 31]. Travel-related expenses and vehicle modifications add to this burden, particularly considering mobility challenges associated with OI [22, 32]. These costs are recurring and often long-term, creating a continuous burden, especially for those with limited or no insurance coverage. Consequently, individuals often resort to charitable fundraising platforms like GoFundMe to alleviate financial strains [22]. However, the success rates of these campaigns vary, with research indicating that campaigns for children tend to fare better compared with those for adults [33]. These findings highlight the need for comprehensive support systems and accessible financial aid for individuals with OI, aiming to alleviate the enduring financial challenges they face.

While OI is associated with substantial resource use, productivity loss and out-of-pocket spending, there remains a need for a more nuanced understanding of the factors driving its economic impact. The current study sheds light on certain attributes within individuals with OI that may drive an increased economic impact, underscoring the importance of future research endeavours. Specifically, further research is needed to understand the disparity in missed workdays between individuals with OI and the general population, explore the underlying factors behind unemployment rate disparities and missed workdays and examine respondent characteristics to grasp productivity challenges in individuals with OI. Additionally, gaining deeper insights into the variations in healthcare resource use within the OI population and conducting country level analyses to gather market-specific cost data are crucial. Exploring specific financial challenges and potential interventions would provide a holistic understanding of the economic impact of OI, guiding targeted strategies to mitigate its effects.

### Strengths and limitations

The IMPACT survey addresses some of the gaps in our knowledge of the economic impact of OI that has rarely been explored in past studies. The large sample size of this dataset enables stratification by individual characteristics. This approach allows us to gain valuable insights into the factors driving healthcare use among individuals with OI.

It is important to note that our study likely underestimated the genuine healthcare needs of individuals with OI due to the timing of the survey, which coincided with the COVID-19 pandemic. The onset of the pandemic significantly influenced healthcare-seeking behaviours, especially for individuals with high-risk conditions like OI. Government and health authorities recommended that people with chronic conditions should minimise exposure to the virus by staying at home [34]. For instance, during the initial UK lockdown in April 2020, HCP visits dropped by 32%, primarily in-person appointments fell from 84% to about 50%, and diagnostic tests reduced to focus on managing COVID-19 cases [35, 36]. While the impact of COVID-19 cannot be fully mitigated within this research, the survey included questions to estimate the effect of the pandemic and better understand limitations of this dataset.

Although this study was open to individuals from all geographies, respondents from North America and Europe are predominantly represented. However, because of the large sample size, unique insights into previously underreported geographies and demographics are possible. Due to the inclusion of a wide range of geographies this dataset includes individuals from multiple healthcare systems and cannot mitigate for varying healthcare pathways, access levels and healthcare provision. Consequently, accurately estimating the typical healthcare-seeking behaviour of adults with OI becomes difficult.

Self-reported data are less robust than registry data and are affected by recall bias; however, the bottom-up approach of collecting patient data allows the capture of costs that are not commonly accounted for, such as out-of-pocket spending and missed workdays. This study did not seek to distinguish healthcare spending specific to OI from healthcare use for any other needs, in part to avoid confounding self-reported data collected within this work further, but this limits our ability to estimate incremental additional costs compared with the overall population.

As an exploratory cost of illness study, our main aim was to offer a snapshot of healthcare resource use and costs among adults with OI. As an international survey designed to reach as large a population as possible with a rare condition, the study was not set up to establish diagnosis-specific costs. Furthermore, market prices were not adjusted to reflect true costs. Despite insights gained from comparisons with past population-based research, the absence of a control group limits the generalisability of our findings, preventing us from capturing incremental costs over time [23].

### Conclusion

IMPACT has generated a novel insight into the healthcare use, productivity losses and out-of-pocket spending of individuals with OI and estimates drivers of increased costs to individuals and society. The analysis of driving factors of healthcare use among individuals with OI underscores the diverse needs within the OI community.

### Supplementary Information

Supplementary Material 1: Appendix Table 1. Dependent and independent variables used in Poisson regression analyses. This table describes the variables included in the Poisson regression analyses.

Supplementary Material 2: Appendix Table 2. Dependent and independent variables used in logistic regression analyses. This table describes the variables included in the logistic regression analyses.

Supplementary Material 3: Appendix Table 3. Dependent and independent variables used in pairwise analyses. This table describes the variables included in the pairwise analyses.

Supplementary Material 4: Appendix Table 4. Demographics by clinical OI type. This table provides the demographics of the population stratified by clinical OI type.

Supplementary Material 5: Appendix Table 5. Demographics by self-reported OI severity. This table provides the demographics of the population stratified by self-reported OI severity.

Supplementary Material 6: Appendix Table 6. Poisson regression analysis of patient-reported characteristics associated with healthcare resource utilisation and cost. This table provides the incidence rate ratios and corresponding 95% confidence intervals derived from Poisson regression analyses of patient reported characteristics associated with healthcare resource utilisation and cost.

Supplementary Material 7: Appendix Table 7. Logistic regression analysis of patient-reported characteristics associated with healthcare resource utilisation and cost. This table provides the odds ratios and corresponding 95% confidence intervals derived from logistic regression analyses of patient reported characteristics associated with healthcare resource utilisation and cost.

Supplementary Material 8: Appendix Figure 1. Incidence rate and odds ratios with 95% confidence intervals for OI severity against healthcare utilisation and costs. These graphs illustrate statistically significant results from 57 different regression analyses, focusing on the relationship between OI severity and healthcare utilisation and costs.

Supplementary Material 9: Appendix Figure 2. Incidence rate and odds ratios with 95% confidence intervals for clinical signs, symptoms and events against healthcare utilisation and costs. These graphs illustrate statistically significant results from 57 different regression analyses, focusing on the relationship between different clinical signs, symptoms and events and healthcare utilisation and costs.

Supplementary Material 10: Appendix Figure 3. Incidence rate and odds ratios with 95% confidence intervals for sex against healthcare utilisation and costs. These graphs illustrate statistically significant results from 57 different regression analyses, focusing on the relationship between sex and healthcare utilisation and costs.

Supplementary Material 11: Appendix Figure 4. Incidence rate and odds ratios with 95% confidence intervals for age and healthcare utilisation and costs. These graphs illustrate statistically significant results from 57 different regression analyses, focusing on the relationship between age and healthcare utilisation and costs.

Supplementary Material 12: Appendix Table 8. Pairwise analysis of mean visits to a generalist over 12 months. This table provides the mean number of visits to a generalist healthcare professional among adults with OI over a 12-month period for the overall population and various subgroups (including sex, OI severity, age and different clinical symptoms and events). The colour coding highlights the variations within subgroups compared with the overall population. Statistically significant results from pairwise analyses are denoted by an asterisk (*).

Supplementary Material 13: Appendix Table 9. Pairwise analysis of mean visits to a specialist over 12 months. This table provides the mean visits to a specialist healthcare professional among adults with OI over a 12-month period for the overall population and various subgroups (including sex, OI severity, age and different clinical symptoms and events). The colour coding highlights the variations within subgroups compared with the overall population. Statistically significant results from pairwise analyses are denoted by an asterisk (*).

Supplementary Material 14: Appendix Table 10. Pairwise analysis of mean visits to a therapist over 12 months. This table provides the mean visits to a therapist healthcare professional among adults with OI over a 12-month period for the overall population and various subgroups (including sex, OI severity, age and different clinical symptoms and events). The colour coding highlights the variations within subgroups compared with the overall population. Statistically significant results from pairwise analyses are denoted by an asterisk (*).

Supplementary Material 15: Appendix Table 11. Pairwise analysis of frequency of hospital and in-patient care use over 12 months. This table provides the mean frequency of hospital and in-patient care use among adults with OI over a 12-month period for the overall population and various subgroups (including sex, OI severity, age and different clinical symptoms and events). The colour coding highlights the variations within subgroups compared with the overall population. Statistically significant results from pairwise analyses are denoted by an asterisk (*).

Supplementary Material 16: Appendix Table 12. Pairwise analysis of frequency of diagnostic tests over 12 months. This table provides the mean frequency of diagnostic tests undergone by adults with OI over a 12-month period for the overall population and various subgroups (including sex, OI severity, age and different clinical symptoms and events). The colour coding highlights the variations within subgroups compared with the overall population. Statistically significant results from pairwise analyses are denoted by an asterisk (*).

Supplementary Material 17: Appendix Table 13. Pairwise analysis of frequency of surgeries in lifetime. This table provides the mean frequency of surgeries undergone by adults with OI over their lifetime for the overall population and various subgroups (including sex, OI severity, age and different clinical symptoms and events). The colour coding highlights the variations within subgroups compared with the overall population. Statistically significant results from pairwise analyses are denoted by an asterisk (*).

Supplementary Material 18: Appendix Table 14. Pairwise analysis of proportion of respondents using consumables and services over 12 months. This table provides the proportions of adults with OI using specific consumables and services over a 12-month period for the overall population and various subgroups (including sex, OI severity, age and different clinical symptoms and events). The colour coding highlights the variations within subgroups compared with the overall population. Statistically significant results from pairwise analyses are denoted by an asterisk (*).

Supplementary Material 19: Appendix Table 15. Pairwise analysis of missed workdays over 4 weeks. This table provides the mean number of workdays missed among adults with OI over a 4-week period for the overall population and various subgroups (including sex, OI severity, age and different clinical symptoms and events). The colour coding highlights the variations within subgroups compared with the overall population. Statistically significant results from pairwise analyses are denoted by an asterisk (*).

Supplementary Material 20: Appendix Table 16. Pairwise analysis of out-of-pocket spending in Euros (€) over 4 weeks. This table provides the mean spend out-of- pocket among adults with OI over a 4-week period for the overall population and various subgroups (including sex, OI severity, age and different clinical symptoms and events). The colour coding highlights the variations within subgroups compared with the overall population. Statistically significant results from pairwise analyses are denoted by an asterisk (*).

Supplementary Material 21: Appendix Table 17. Pairwise analysis *p*-values for visits to a healthcare professional over 12 months. This table provides a comprehensive overview of *p*-values obtained from pairwise analyses of patient reported characteristics associated with visits to various healthcare professionals over a 12-month period.

Supplementary Material 22: Appendix Table 18. Pairwise analysis *p*-values for visits to the hospital and in-patient care use over 12 months. This table provides a comprehensive overview of *p*-values obtained from pairwise analyses of patient reported characteristics associated with visits to the hospital and in-patient care use over a 12-month period.

Supplementary Material 23: Appendix Table 19. Pairwise analysis *p*-values for diagnostic tests undertaken over 12 months. This table provides a comprehensive overview of *p*-values obtained from pairwise analyses of patient reported characteristics associated with diagnostic tests undertaken over a 12-month period.

Supplementary Material 24: Appendix Table 20. Pairwise analysis *p*-values for OI surgeries undertaken in lifetime. This table provides a comprehensive overview of *p*-values obtained from pairwise analyses of patient reported characteristics associated with surgeries undertaken in an adult with OI’s lifetime.

Supplementary Material 25: Appendix Table 21. Pairwise analysis *p*-values for the usage of OI consumables and services over 12 months. This table provides a comprehensive overview of *p*-values obtained from pairwise analyses of patient reported characteristics associated with the use of specific OI consumables and services over a 12-month period.

Supplementary Material 26: Appendix Table 22. Pairwise analysis *p*-values for missed workdays over 4 weeks. This table provides a comprehensive overview of *p*-values obtained from pairwise analyses of patient reported characteristics associated with missed workdays over a 4-week period.

Supplementary Material 27: Appendix Table 23. Pairwise analysis *p*-values for out-of-pocket spending over 4 weeks. This table provides a comprehensive overview of *p*-values obtained from pairwise analyses of patient reported characteristics associated with out-of-pocket spending over a 4-week period.

## Data Availability

The data that support the findings of this study are not openly available due to reasons of sensitivity. They are managed by a data management committee and are available upon reasonable request to the authors.
