# Supplementary material for: The IMPACT Survey: the economic impact of osteogenesis imperfecta in adults
Source: Orphanet J Rare Dis. 2024 Jun 3;19:222. doi: 10.1186/s13023-024-03218-6 (PMC11149192; doi:10.1186/s13023-024-03218-6)
Supplement: Supplementary file 1 — Supplementary Material 1: Appendix Table 1. Dependent and independent variables used in Poisson regression analyses. This table describes the variables included in the Poisson regression analyses. [file 13023_2024_3218_MOESM1_ESM.docx]

Appendix Table 1 Dependent and independent variables used in Poisson regression analyses

| Dependent variables | **Frequency of healthcare professional visits in the past 12 months** (including general practitioners/family doctors, nurse practitioners/care coordinators, paediatricians, orthopaedic surgeons/orthopaedists, nutritionists, psychotherapists/counsellors, physiotherapists, dentists/orthodontists, audiologists, ophthalmologists, gynaecologists/obstetricians, endocrinologists, cardiologists, neurologists, gastroenterologists, rheumatologists, pulmonologists, rehabilitation therapists/doctors, and occupational therapists) |
| --- | --- |
|  | **Frequency of hospital and in-patient care use in the past 12 months** (including hospital visits, emergency room visits, nights in hospital, and nights in rehabilitation) |
|  | **Frequency of diagnostic tests in the past 12 months** (including blood tests, urine tests, X-rays, computerised tomography scans, bone density scans, magnetic resonance imaging scans, echocardiograms, and audiology tests) |
|  | **Frequency of OI-related surgeries in an individual's lifetime** (including rodding, fracture repairs, spinal, hearing, dental, heart, basilar invagination, and soft tissue) |
|  | **Missed workdays in the past 4 weeks** |
|  | **Out-of-pocket spending in the past 4 weeks** (including medicines, physiotherapy, psychotherapy, travel to medical appointments, personal care/support assistance and the total spend of queried categories) |
| Independent variables | **Age** |
|  | **Sex** |
|  | **Self-reported OI severity** |
|  | **Clinical signs, symptoms and events experienced in the past 12 months** (including pain, fractures [including arm, leg, vertebral and rib], fatigue, scoliosis or other bone problems, soft tissue problems, hypermobility, joint problems, hearing problems, vision problems, dental problems, breathing problems, stomach problems, kidney problems, high blood pressure, sexual problems, sleep disturbances, mental health problems, basilar invagination, gynaecological problems, fertility problems, chewing problems, obesity, and low weight) |
