# Supplementary material for: The IMPACT Survey: the economic impact of osteogenesis imperfecta in adults
Source: Orphanet J Rare Dis. 2024 Jun 3;19:222. doi: 10.1186/s13023-024-03218-6 (PMC11149192; doi:10.1186/s13023-024-03218-6)
Supplement: Supplementary file 2 — Supplementary Material 2: Appendix Table 2. Dependent and independent variables used in logistic regression analyses. This table describes the variables included in the logistic regression analyses. [file 13023_2024_3218_MOESM2_ESM.docx]

Appendix Table 2 Dependent and independent variables used in logistic regression analyses

| Dependent variables | **OI consumables and services used in the past 12 months** (including manual wheelchair, powered wheelchair, walking aids, hearing aids, breathing aids, home modifications, vehicle modifications, work modifications, personal care/support assistance and dental work) |
| --- | --- |
| Independent variables | **Age** |
|  | **Sex** |
|  | **Self-reported OI severity** |
|  | **Clinical signs, symptoms and events experienced in the past 12 months** (including pain, fractures [including arm, leg, vertebral and rib], fatigue, scoliosis or other bone problems, soft tissue problems, hypermobility, joint problems, hearing problems, vision problems, dental problems, breathing problems, stomach problems, kidney problems, high blood pressure, sexual problems, sleep disturbances, mental health problems, basilar invagination, gynaecological problems, fertility problems, chewing problems, obesity, and low weight) |
