# Supplementary material for: The IMPACT Survey: the economic impact of osteogenesis imperfecta in adults
Source: Orphanet J Rare Dis. 2024 Jun 3;19:222. doi: 10.1186/s13023-024-03218-6 (PMC11149192; doi:10.1186/s13023-024-03218-6)
Supplement: Supplementary file 4 — Supplementary Material 4: Appendix Table 4. Demographics by clinical OI type. This table provides the demographics of the population stratified by clinical OI type. [file 13023_2024_3218_MOESM4_ESM.docx]

Appendix Table 4 Demographics by clinical OI type

|  | **Type 1 (n=543) ^a,b^** | **Type 3 (n=225) ^a,b^** | **Type 4 (n=158) ^a,b^** |
| --- | --- | --- | --- |
| Age, mean (range) ^c^ | 44.2 (18─85) | 37.8 (18─77) | 44.7 (19─76) |
| **Geography, N (%) ^d^** | | | |
| Europe | 350 (64.5) | 137 (60.9) | 95 (60.1) |
| North America | 137 (25.2) | 57 (25.3) | 45 (28.5) |
| South America | 12 (2.2) | 21 (9.3) | 6 (3.8) |
| Asia | 23 (4.2) | 9 (4.0) | 5 (3.2) |
| Africa | 0 (0.0) | 0 (0.0) | 0 (0.0) |
| Australia/Oceania | 21 (3.9) | 1 (0.4) | 7 (4.4) |
| **Self-reported OI severity, N (%) ^a,e^** | | | |
| Mild | 322 (59.3) | 6 (2.7) | 29 (18.4) |
| Moderate | 202 (37.2) | 113 (50.2) | 100 (63.3) |
| Severe | 14 (2.6) | 99 (44.0) | 24 (15.2) |
| I don’t know or prefer not to say | 5 (0.9) | 7 (3.1) | 5 (3.2) |
| **Employment status, N (%) ^f^** | | | |
| Not in paid employment | 194 (35.7) | 101 (44.9) | 76 (48.1) |
| Employed full time | 207 (38.1) | 61 (27.1) | 52 (32.9) |
| Employed part time | 90 (16.6) | 45 (20.0) | 23 (14.6) |
| Self-employed | 46 (8.5) | 17 (7.6) | 6 (3.8) |
| Other ^g^ | 4 (0.7) | 1 (0.4) | 1 (0.6) |
| Prefer not to say | 2 (0.4) | 0 (0) | 0 (0) |

Abbreviations: OI, osteogenesis imperfecta

Footnotes: ^a^ Also reported in Westerheim et al. 2024; ^b^ Question 17 " If you have received an OI type as part of your OI diagnosis or treatment, please indicate your type using the dropdown below”; ^c^ Question 1 “What is your age?”; ^d^ Question 7 “What is your country of residence?”; ^e^ Question 18 “How would you describe the severity of your OI?”; ^f^ Question 9 and 10 “Please indicate which of the following best describe you/What is your current paid employment status?”; ^g^ ‘Other’ includes respondents who were in paid full-time internships or paid jobs but were not working at the time due to a leave of absence
