# Supplementary material for: The IMPACT Survey: the economic impact of osteogenesis imperfecta in adults
Source: Orphanet J Rare Dis. 2024 Jun 3;19:222. doi: 10.1186/s13023-024-03218-6 (PMC11149192; doi:10.1186/s13023-024-03218-6)
Supplement: Supplementary file 5 — Supplementary Material 5: Appendix Table 5. Demographics by self-reported OI severity. This table provides the demographics of the population stratified by self-reported OI severity. [file 13023_2024_3218_MOESM5_ESM.docx]

Appendix Table 5 Demographics by self-reported OI severity

|  | **Mild OI (n=507) ^a,b^** | **Moderate OI (n=671) ^a,b^** | **Severe OI (n=205) ^a,b^** |
| --- | --- | --- | --- |
| Age, mean (range) ^c^ | 43.1 (18–83) | 43.7 (18–85) | 42.3 (18–76) |
| **Geography, N (%) ^d^** | | | |
| Europe | 316 (62.3) | 417 (62.1) | 139 (67.8) |
| North America | 130 (25.6) | 175 (26.1) | 38 (18.5) |
| South America | 12 (2.4) | 29 (4.3) | 17 (8.3) |
| Asia | 25 (4.9) | 33 (4.9) | 10 (4.9) |
| Africa | 3 (0.6) | 3 (0.5) | 0 (0.0) |
| Australia/Oceania | 21 (4.1) | 14 (2.1) | 1 (0.5) |
| **OI type, N (%) ^e^** | | | |
| Type 1 | 322 (63.5) | 202 (30.1) | 14 (6.8) |
| Type 3 | 6 (1.2) | 113 (16.8) | 99 (48.3) |
| Type 4 | 29 (5.7) | 100 (14.9) | 24 (11.7) |
| Other | 60 (11.8) | 125 (18.6) | 28 (13.7) |
| I don’t know or prefer not to say | 90 (17.8) | 131 (19.5) | 40 (19.5) |
| **Employment status, N (%) ^f^** | | | |
| Not in paid employment | 157 (31.0) | 304 (45.31) | 104 (50.7) |
| Employed full time | 221 (43.6) | 215 (32.0) | 49 (23.9) |
| Employed part time | 81 (16.0) | 100 (14.9) | 38 (18.5) |
| Self-employed | 45 (8.9) | 43 (6.4) | 14 (6.8) |
| Other ^g^ | 2 (0.4) | 5 (0.8) | 0 (0.0) |
| Prefer not to say | 1 (0.2) | 4 (0.6) | 0 (0.0) |

Abbreviations: OI, osteogenesis imperfecta

Footnotes: ^a^ Also reported in Westerheim et al. 2024; ^b^ Question 18 “How would you describe the severity of your OI?”; ^c^ Question 1 “What is your age?”; ^d^ Question 7 “What is your country of residence?”; ^e^ Question 17 " If you have received an OI type as part of your OI diagnosis or treatment, please indicate your type using the dropdown below”; ^f^ Question 9 and 10 “Please indicate which of the following best describe you/What is your current paid employment status?”; ^g^ ‘Other’ includes respondents who were in paid full-time internships or paid jobs but were not working at the time due to a leave of absence
